# The education word gap emerges by 18months: findings from an Australian prospective study

**DOI:** 10.1186/s12887-021-02712-1

**Published:** 2021-05-21

**Authors:** Mary E. Brushe, John Lynch, Sheena Reilly, Edward Melhuish, Murthy N. Mittinty, Sally A. Brinkman

**Affiliations:** 1 Telethon Kids Institute, University of Western Australia, Level 15, 31 Flinders St, Adelaide, South Australia 5000 Australia; 2 School of Public Health, University of Adelaide, Level 9, Adelaide Health & Medical Science Building, 57 North Terrace, Adelaide, South Australia 5005 Australia; 3 Population Health Sciences, Bristol Medical School, University of Bristol, First Floor, 5 Tyndall Avenue, Bristol, B28 1UD UK; 4 Menzies Health Institute Queensland G40 Griffith Health Centre, Griffith University, Level 8.86 Gold Coast Campus, Southport, Queensland 4222 Australia; 5 Department of Education, University of Oxford, 15 Norham Gardens, Oxford, OX2 6PY UK

**Keywords:** Word gap, Parent talk, Inequality, Early childhood development, Language

## Abstract

**Background:**

The idea of the 30 million word gap suggests families from more socioeconomically advantaged backgrounds engage in more verbal interactions with their child than disadvantaged families. Initial findings from the Language in Little Ones (LiLO) study up to 12months showed no word gap between maternal education groups.

**Methods:**

Families with either high or low maternal education were purposively recruited into a five-year prospective study. We report results from the first three waves of LiLO when children were 6, 12 and 18months old. Day-long audio recordings, obtained using the Language Environment Analysis software, provided counts of adult words spoken to the child, child vocalizations and conversational turns.

**Results:**

By the time children were 18months old all three measures of talk were 0.5 to 0.7 SD higher among families with more education, but with large variation within education groups. Changes in talk from 6 to 18months highlighted that families from low educated backgrounds were decreasing the amount they spoke to their children (4219.54, 95% CI -6054.13, 2384.95), compared to families from high educated backgrounds who remained relatively stable across this age period (369.13, 95% CI 2344.57, 1606.30).

**Conclusions:**

The socioeconomic word gap emerges between 12 and 18months of age. Interventions to enhance maternal communication, child vocalisations and vocabulary development should begin prior to 18months.

**Supplementary Information:**

The online version contains supplementary material available at 10.1186/s12887-021-02712-1.

## Background

The emergence of socioeconomic inequalities in many areas of childrens health and development is evident early in life [1, 2]. Understanding when and how these inequalities develop is a key question for researchers and policymakers because preventive interventions should be in place before health and development gaps become entrenched [3]. Hart and Risley famously coined the term 30 million word gap by estimating through linear extrapolation of data collected from 10 to 36months, that by age four, parents in the United States (US) who were on welfare had spoken 30 million words less to their child than parents with professional occupations [4]. In a 10-year follow up, they found these socioeconomic differences predicted subsequent verbal ability, receptive and expressive vocabulary, and academic achievement in grade 3 [5].

The term 30 million word gap has garnered enormous attention, with over 113 million google hits. In response, new technology has been developed [6] and considerable resources expended on initiatives across the world aiming to reduce the word gap. The Hart and Risley findings were based on a convenience sample of 42 families in Kansas, with only 6 families in the welfare category, compared to 13 families in the professional category and 23 families in the working class category. Furthermore, data were collected through researchers videotaping 1h in the familys home per month, which may not be representative of the natural home environment. Language data were collected from 10months of age onwards, limiting the understanding of critical language experiences during the first year of life. The validity and generalizability of Hart and Risleys findings have been widely debated [7,10].

Gilkerson and colleagues [11] attempted to overcome some of the limitations of Hart and Risleys work through the use of newly developed speech recognition technology, Language Environment Analysis (LENA). Researchers were able to objectively measure a familys home language environment to capture the number of words children heard over a day. The study involved 329 English speaking families with children aged between 2 and 48months, from Denver. Families completed LENA recording days once a month for 6 months and a subset of 59 families completed monthly recording days for an additional 32months. Their results estimated a 4-million-word gap by age 4 between mothers with some high school vs. those with a college degree.

The Language in Little Ones (LiLO) study is a prospective study of Australian families aiming to understand maternal education differences in the number of words children hear and speak in the home environment during the first 5 years of life. The LiLO study started collecting language data in the home, involving day-long recordings, when the children were 6months old with data collection occurring every 6 months, until their first year of schooling, around age 5. We previously reported that when children were 6 and 12months old there were no meaningful differences in any measure of parent-child talk between maternal education groups [12]. There was large variability, with high and low talkers within both education groups.

The present study includes new data from the LiLO study when children were 18 months old. This is an important age in childrens language development when they are beginning to expand their vocabularies. Here we report all data currently available from the LiLO study including the number of adult words spoken to the child, number of child vocalizations and number of conversations between adult and child over a day when the children are 6, 12, and 18months old by levels of maternal education.

## Methods

### Study design

The LiLO study commenced recording parent-child talk when children were 6months old, with repeated measures every 6 months until child age 5 years. LiLO was explicitly designed to maximize contrasts across maternal education groups, by stratifying recruitment into a low educated group (mothers without any post-secondary school qualifications), and a high educated group (mothers with a bachelors degree at minimum). At each wave of data collection, families undertook day-long (16-h) audio recordings. A $10 supermarket voucher was provided to families as compensation after each wave.

### Participants

Recruitment occurred within Adelaide and Port Pirie in South Australia, Bunbury in Western Australia and the Gold Coast in Queensland between April 1, 2017 and July 31, 2019. Expecting mothers were approached at public hospitals while waiting for their antenatal appointments. Additionally, postnatal recruitment occurred at Child and Family Health Services during early parenting groups and drop-in clinics in Adelaide, Port Pirie and Bunbury. Mothers were also approached at local shopping centres, council-run immunization clinics, community playgroups, childrens centres and libraries across all locations. Families were excluded if they did not speak English in the home or ifthe mothers level of education did not fall within the low or high educated categories. They were also excluded if their child was part of a multiple birth, was born premature (<37weeks), had a diagnosed cause of language impairment (e.g. hearing impairment, Down Syndrome, Cerebral Palsy) or was born outside the date range of January 1, 2017 and December 31, 2017.

**Fig. 1:**
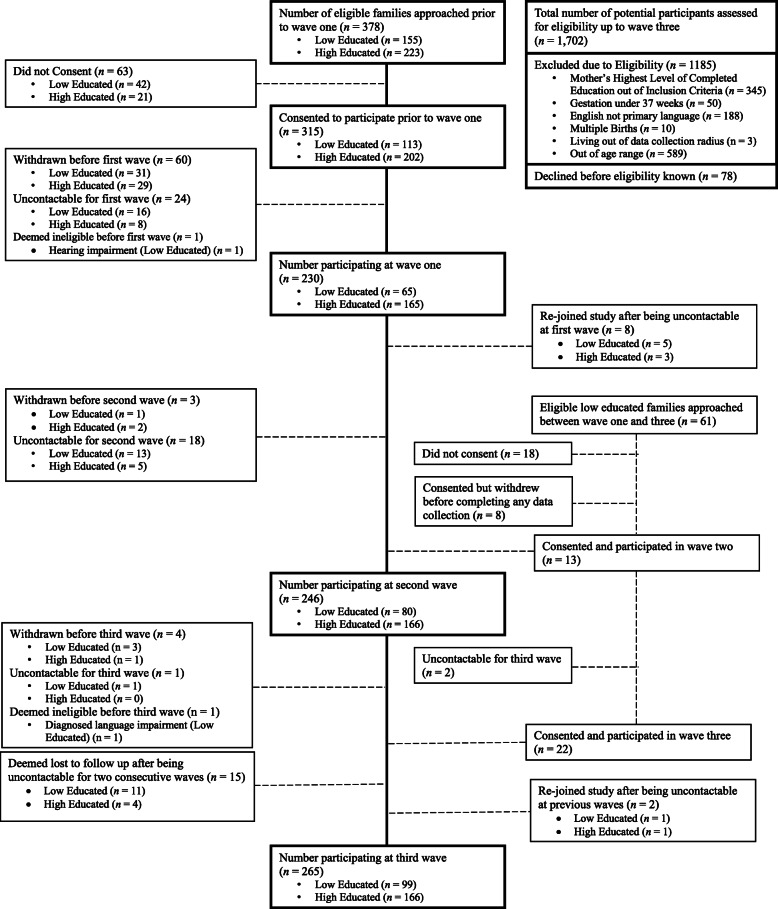
Flow chart of recruitment numbers

Figure1 provides a detailed flow chart of participant numbers across the first three waves. As is common in prospective studies, there was difficulty in attempting to recruit socioeconomically disadvantaged groups [13]. At the first wave, only 65 low educated families were participating in the study despite extensive and exhaustive recruitment efforts. To boost sample size among the low educated, we extended the recruitment timelines and locations which meant families could join the longitudinal study even if they had missed the first or second wave of data collection. An additional 35 low educated families joined the study and only seven families (4 low educated; 3 high educated) had withdrawn since the study started. A home visit occurred with each family within 2 months of the childs 6, 12 and 18month birthdates. Data collection procedures have been previously described, with processes remaining consistent at each wave for all families in the study [12].

### Measures

The Language Environment Analysis (LENA) system was used to capture the childs home language environment. The LENA technology comprises a digital language processor (DLP) and LENA computer software to automatically process the audio captured through the DLP using algorithmic analysis of the acoustic properties in the speech signal [6, 14, 15]. Three key LENA measures were used in this study: adult word count (AWC; the number of adult words spoken to the child), child vocalization count (CVC; the number of speech-related sounds made by the child) and conversational turn count (CT; the number of alternations between adult and child occurring within at least 5s of each other). Home activity diaries were also completed by the parents outlining the activities of the child, by the hour, throughout the recording day. Total word counts, from the LENA software, were used in the analysis when the full 16-h recording was completed or the activity diaries confirmed that the LENA device was turned off when the child went to sleep. Adjusted word counts were calculated if the LENA device was turned off prior to childs bedtime, whereby average hourly counts were added to the total reported word count to take the total recording time up until the child fell asleep, as reported by the parents in the home activity diaries. Adjusted word counts were only used for one low educated family in wave 1 and one high educated family in wave 3. Reliability testing by the LENA Foundation has reported high levels of agreement between human transcribers and LENA system classification [15].

### Statistical approach

**Fig. 2:**
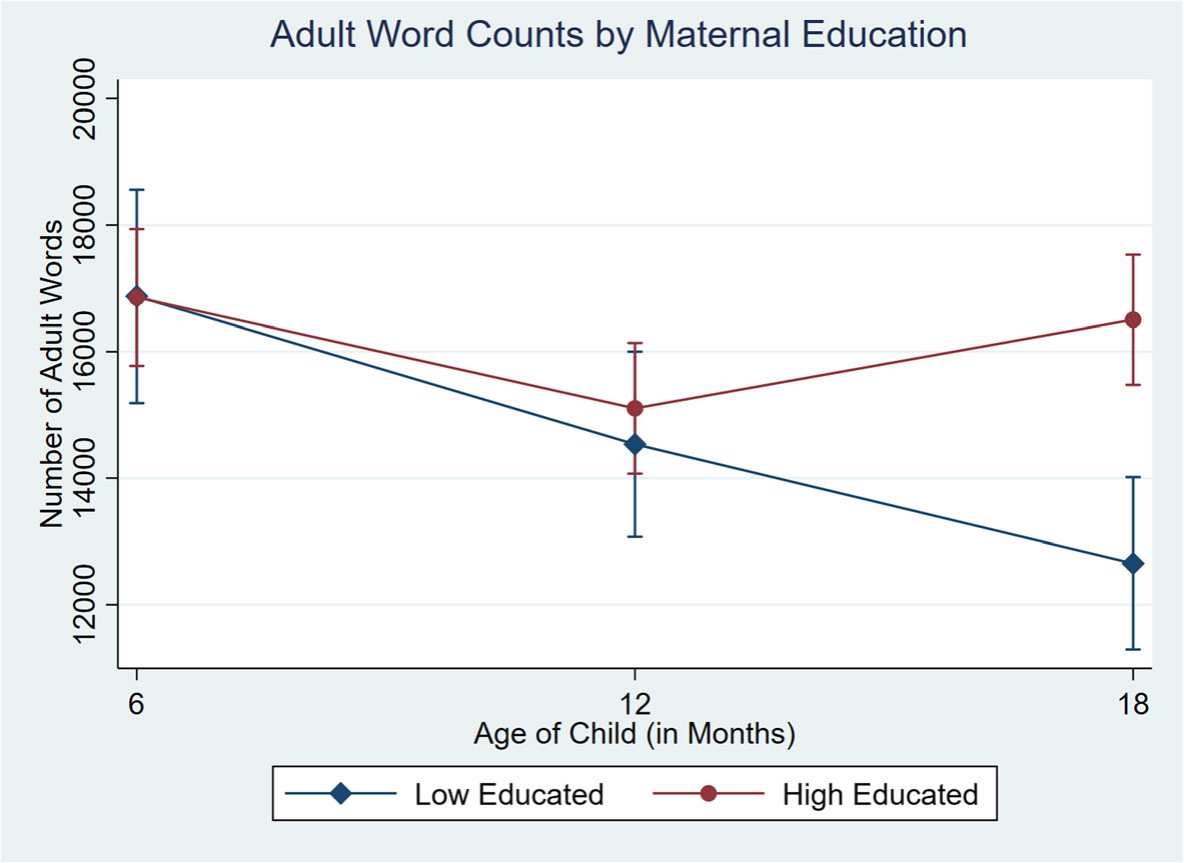
Predicted mean adult word count and 95% CI by maternal education across 6month, 12month and 18month wave of data collection

**Fig. 3:**
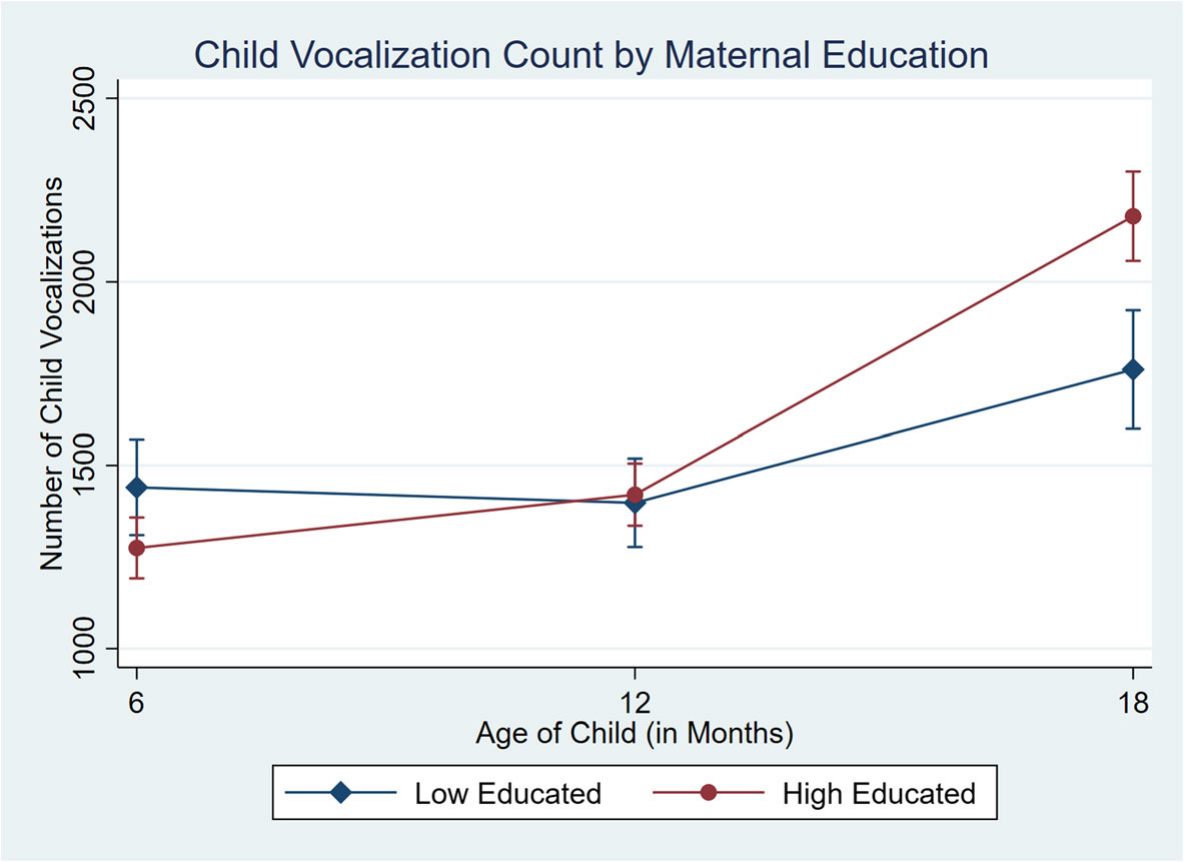
Predicted mean child vocalizations count and 95% CI by maternal education across 6month, 12month and 18month wave of data collection

**Fig. 4:**
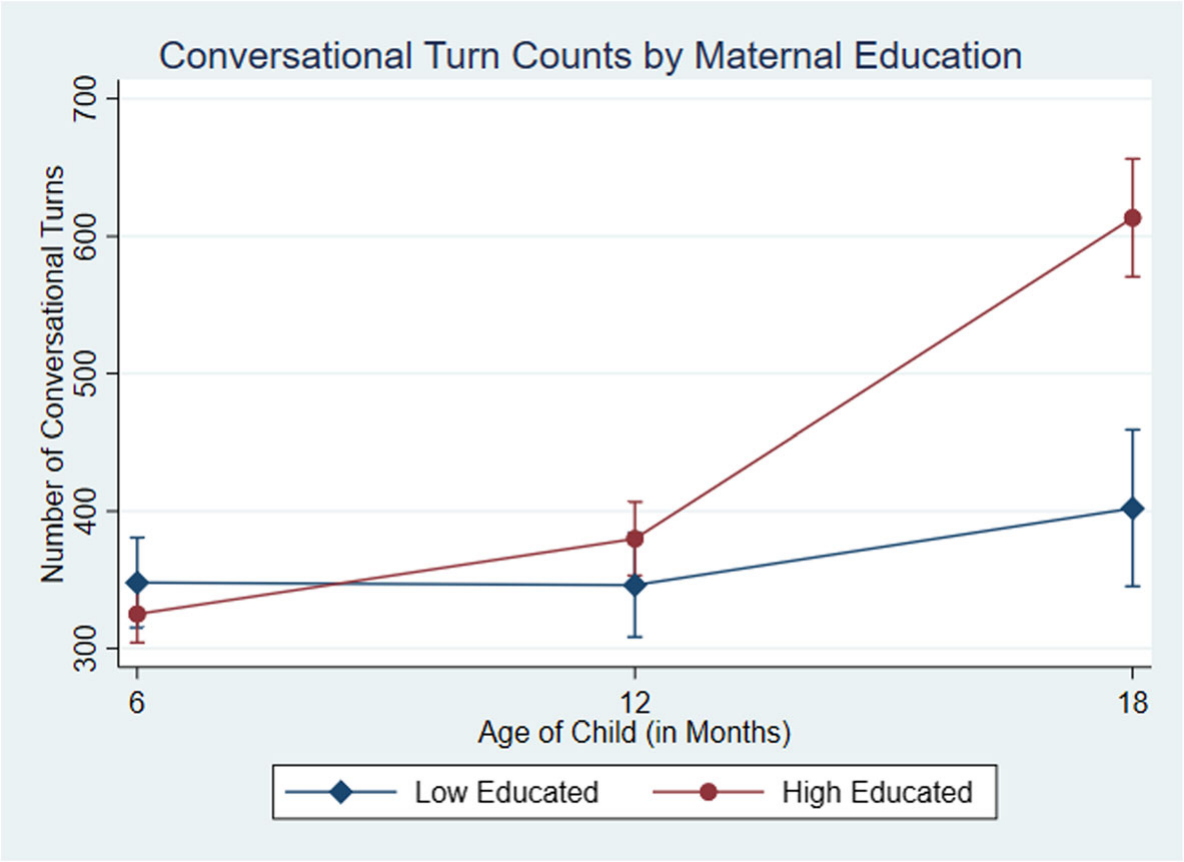
Predicted mean conversational turns count and 95% CI by maternal education across 6month, 12month and 18month wave of data collection

Parent-child talk variables were modelled using random effects longitudinal models using the *xtmixed* command in Stata, to understand changes in adult word counts, child vocalizations count, and conversational turn counts according to maternal education, from child ages 6, 12 and 18months old. The interaction of mothers education and wave of data collection was included as the only predictor in the model to identify how changes over time differ between education groups. The parameters were computed using the expectation maximisation (EM) algorithm. To identify differences between maternal education groups and their word counts across each wave, we used the *margins* command in Stata to calculate the predicted means for low and high educated families at each time point and plotted their mean word counts and 95% confidence intervals across waves in Fig. 2, 3 and 4. A comparison of means from the observed data and the computed model is provided in the supplementary appendices (See Additionalfile1). Effect sizes were also calculated using Cohens *d* [16].

To ensure the addition of the extra 35 low educated families at waves 2 and 3 did not affect the results, we undertook a sensitivity analysis that only included families who had participated since wave 1. Details of the sensitivity analyses are also provided in the supplementary appendices (See Additionalfile2). All analyses and figures were conducted using Stata version 16 [17].

## Results

Data for the first three waves were collected between August 1, 2017 and July 31, 2019. Of those families actively participating in the study, LENA data was not available for 11 families across the three waves. This was due to nine families at wave three skipping their visit due to personal reasons and for two families the LENA device malfunctioned (one at wave 1 and one at wave 3). One family was deemed ineligible due to a diagnosed cause of language impairment at the third wave, so their data was retrospectively removed.

The sample varied slightly across waves due to the increase in participant numbers, as shown in Table1. The final analysis sample consisted of 163 families in the high educated group and 92 families in the low educated group. The average age of the mother at childbirth was 31.28years and 85% of mothers were employed prior to their pregnancy. Just over half the children in the sample were first born and 54% were female.

**Table 1:** Sociodemographic Characteristics of the Sample

	6month Data Collection (***N***=228)	12month Data Collection (***N***=245)	18month Data Collection (***N***=255)
Child			
Age, mo, mean (SD)	5.82 (0.58)	11.99 (0.51)	18.02 (0.56)
Girls, n (%)	122 (53)	130 (53)	136 (53)
Gestation, wk., mean (SD)	39.1 (1.35)	39.18 (1.50)	39.19 (1.49)
Firstborn, n (%)	128 (56)	131 (53)	136 (53)
Mother			
Highest level of completed education, University, n (%)	164 (72)	166 (68)	163 (64)
Age at childbirth, y, mean (SD)	31.36 (4.42)	31.22 (4.57)	31.28 (4.84)
Working up until pregnancy, yes, n (%)	199 (87)	211 (86)	217 (85)

Table 2 shows the results from the random effects model that estimates the interaction between mothers education and wave of data collection on the three LENA measures: adult word counts, child vocalization counts and conversational turn counts. The coefficient demonstrates the changes in growth for both low and high educated groups as compared to the 6month baseline for the low educated group. As can be noted from the model, for adult word counts, families from low educated backgrounds were talking 4219.54 words less, 95% CI (6054.13, 2384.95) to their children by 18months, compared to high educated mothers who remained relatively stable across waves with only 369.13 fewer words 95% CI (2344.57, 1606.30) by 18months. For child vocalization counts, the model demonstrates children from both the low (320.74, 95% CI 126.61, 514.88) and high (739.04, 95% CI 560.66, 917.53) educated groups increased their number of vocalizations by 18months, but high educated children grew their vocalizations at a faster rate. For conversational turn counts, both high and low educated families had little growth between the first and second wave, however between the ages of 12 and 18months, growth in turns between adult and child for the high educated group (265.62, 95% CI 211.52, 319.73) exceeded that of the low educated group (54.22, 95% CI -5.54, 113.98). The 95% confidence intervals in the models highlight large variability in growth across waves for both groups. However, on average low educated adults are talking less to their children by 18months.

**Table 2:** Random effects model estimates for LENA measures across maternal education groups

	Coef.	*p*	95% CI	
**Adult Word Counts**				
*Number of adult words at 6months among low educated=16,872.86*				
Low Educated at 12months	-2336.90	0.016	-4243.32,	430.48
Low Educated at 18months	4219.54	0.000	6054.13,	2384.95
High Educated at 6months	16.64	0.987	2019.84,	1986.55
High Educated at 12months	1768.77	0.080	3746.90,	209.36
High Educated at 18months	369.13	0.714	2344.57,	1606.30
**Child Vocalisations Counts**				
*Number of child vocalizations at 6months among low educated=1440.28*				
Low Educated at 12months	42.15	0.553	181.41,	97.11
Low Educated at 18months	320.74	0.001	126.61,	514.88
High Educated at 6months	165.36	0.036	319.98,	10.74
High Educated at 12months	19.97	0.801	175.49,	135.54
High Educated at 18months	739.04	0.000	560.55,	917.53
**Conversational Turn Counts**				
*Number of conversational turns at 6months among low educated=347.90*				
Low Educated at 12months	1.79	0.935	44.76,	41.16
Low Educated at 18months	54.22	0.075	5.54,	113.98
High Educated at 6months	22.92	0.248	61.82,	15.98
High Educated at 12months	31.99	0.139	10.42,	74.41
High Educated at 18months	265.62	0.000	211.52,	319.73

The graphs in Figs.2, 3 and 4 depict the predicted mean and 95% confidence intervals for each measure of talk by maternal education groups at 6, 12 and 18months of age. The figures show the emergence of the word gap for the number of adult words, child vocalizations and conversational turns by the time children were 18months old, in line with the results of the random effects model. For adult words spoken (Fig. 2) we found a difference of 17 words at 6months, 568 words at 12months and 3851 words at 18months, with families in the high educated group talking more at wave two and three. For child vocalizations (Fig.3) children from the low educated group were vocalizing slightly more, with a difference between groups of 166 vocalizations at 6months. By 12months, there were only 22 more vocalizations on average from children of high educated mothers and by 18months, children in the high educated group had on average 418 more vocalizations. For conversational turns (Fig.4), there were similar differences at 6 and 12months with 24 and 34 turns between adult and child respectively. Families in the low educated group engaged in slightly more conversational turns at 6months but families in the high educated group had more conversations at 12months. Similarly, as with the adult words and child vocalizations, by 18months the difference in conversational turns had grown to 212 turns with more in the highly educated group. Effects for mothers with higher education ranged from 0.5 SD for child vocalizations up to 0.7 SD for conversational turns. Sensitivity analysis that included only families who were observed at each time point did not change the results (See Additional file 1).

## Discussion

These results demonstrate that the word gap between high and low educated mothers emerges between 12 and 18months. The differences between high and low educated mothers were seen for adult words, child vocalizations and conversational turns with effects ranging from 0.5 for word counts to 0.7 SD for conversational turns. As well as understanding the emergence of mean differences in all measures of talk by 18months, it is important to note the large variability within education groups. There are high and low talkers across the socioeconomic spectrum even though on average more educated mothers engaged in more talk. These results are generally consistent with Gilkerson et al. [11], who reported more talk among high educated mothers in the aggregated age band from 20 to 26months.

When considering the implementation of interventions to support the home language environments of infants and toddlers, these results suggest a proportionate universalist approach [18] may be more appropriate, whereby services are universally available but designed with a scale and intensity that is proportionate to the nature of disadvantage. While there is a mean difference between education groups at 18months, there is also large variability in parent-child talk in both education groups, hence targeting interventions only towards low educated families would miss a large proportion of children who are experiencing lower levels of language stimulation in the home among better educated mothers. Targeting of interventions to particular sub-populations presents challenges in reducing inequalities in early childhood development [2].

A limitation of the current study is the differences in sample size across the education groups, with fewer families participating in the low educated group than originally planned. Nonetheless, the low educated group is 10 times larger than Hart and Risleys welfare group and twice as large as Gilkersons et al. some high school group. Numerous strategies were employed to encourage participation. However fewer mothers were identified as eligible in this group resulting in a lower recruitment rate. At later waves an additional 35 families were recruited to the low education group despite missing early waves of data collection and recruitment will continue until our target sample size is reached.

The findings provide support for the existence of a socioeconomic word gap and that this gap emerges between 12 and 18months of age. However, longer term data are required to quantify the size of the word gap by age 4. Key strengths of the LiLO study are that data collection began when children were 6months old and it captures day-long audio recordings, compared to Hart and Risley who only captured 1-h of data in the early evening and did not begin data collection until 10-months old. Each family in the LiLO study is also followed longitudinally, unlike only the small subset of families from the Gilkerson et al. study. Additionally, the larger sample, compared to both Hart and Risley and Gilkerson et al., and the use of the LENA technology means LiLO is well placed to continue quantifying the socioeconomic disparities in talk during the first 5 years of life. Importantly, data were from a population-based sample purposively designed to maximise education exposure contrasts as has been recommended by leading methodologists [19]. These results are likely to be generalizable to the English-speaking Australian population, and probably other English-speaking populations, although there may be ethnic and cultural differences that were not examined in this study. Future LiLO research will consider whether trajectories of talk influence later developmental outcomes and how this differs for maternal education groups. It will also be important to monitor the large variation within the two education groups to see if it is maintained as children age, and if an environment of high talking among low educated families is associated with better child development outcomes.

## Conclusion

These results from the LiLO study suggest a socioeconomic word gap emerges between the ages of 12 and 18months. Families from low educated backgrounds decreased the amount they spoke to their children between 6 and 18months, compared to families from high educated backgrounds whose quantity of talk remained relatively stable across the same period. This is the first study to have used an objective measure of a childs home language environment and been able to provide insight into the timing of the divergence of parent-child talk between maternal education groups. This finding suggests the implementation of proportionate universal programs that encourage parents to talk more to their child should occur prior to 18months of age.

## Supplementary Information

**Additional file 1: Supplementary Appendix.** Observed Means vs Computed Means. To compare the observed means in the raw data and the predicted means computed using the *margins* command.

**Additional file 2: Supplementary Appendix.** Sensitivity Analysis. To ensure the addition of the extra 35 low educated families at waves 2 and 3 did not affect the results, we undertook a sensitivity analysis which only included families who had participated since wave 1.

## Data Availability

The datasets generated and analysed during the current study are not publicly available due to lack of informed consent for data sharing at the time of collection, but are available from the corresponding author on reasonable request. For further information on the data and materials used in this study, please contact the corresponding author.

## Abbreviations

LENA: Language ENvironment Analysis
LiLO: Language in Little Ones
DLP: Digital Language Processer
AWC: Adult Word Count
CVC: Child Vocalization Count
CT: Conversational Turn
