# Supplementary material for: The education word gap emerges by 18months: findings from an Australian prospective study
Source: BMC Pediatr. 2021 May 21;21:247. doi: 10.1186/s12887-021-02712-1 (PMC8139043; doi:10.1186/s12887-021-02712-1)
Supplement: Supplementary file 1 — Additional file 1: Supplementary Appendix. Observed Means vs Computed Means. To compare the observed means in the raw data and the predicted means computed using the margins command. [file 12887_2021_2712_MOESM1_ESM.docx]

**Supplementary Appendix 1**

**Observed Means vs. Predicted Means.**

Predicted means for adult words, child vocalizations and conversational turns were computed by adding or subtracting the coefficient from the intercept estimate to reflect their margins. The intercept estimate uses the refence category of the low educated group at wave 1. As an example, the predicted mean for low educated mothers at wave 2 was computed as 16,872.86 – 2,336.898 = 14,535.96. This data was then used to create Figure 2 – 4. A comparison of the means from the observed data and the margins computed from the model are presented in Table S1. This demonstrates there is very little difference between the observed mean and the predicted mean on all three LENA measures.

| **Table S1. Comparison of observed means and predicted means for LENA measures** | | |
| --- | --- | --- |
|  | Observed Data | Computed Model |
| **Adult Word Counts** |  |  |
| Low Educated at 6 months | 16,768 | 16,873 |
| Low Educated at 12 months | 14,407 | 14,536 |
| Low Educated at 18 months | 12,692 | 12,653 |
| High Educated at 6 months | 16,884 | 16,856 |
| High Educated at 12 months | 15,118 | 15,104 |
| High Educated at 18 months | 16,521 | 16,504 |
| **Child Vocalisations Counts** |  |  |
| Low Educated at 6 months | 1,447 | 1,440 |
| Low Educated at 12 months | 1,407 | 1,398 |
| Low Educated at 18 months | 1,772 | 1,761 |
| High Educated at 6 months | 1,268 | 1,274 |
| High Educated at 12 months | 1,421 | 1,420 |
| High Educated at 18 months | 2,177 | 2,179 |
| **Conversational Turn Counts** |  |  |
| Low Educated at 6 months | 348 | 348 |
| Low Educated at 12 months | 346 | 346 |
| Low Educated at 18 months | 406 | 402 |
| High Educated at 6 months | 324 | 324 |
| High Educated at 12 months | 380 | 379 |
| High Educated at 18 months | 612 | 614 |
