# Supplementary material for: The education word gap emerges by 18months: findings from an Australian prospective study
Source: BMC Pediatr. 2021 May 21;21:247. doi: 10.1186/s12887-021-02712-1 (PMC8139043; doi:10.1186/s12887-021-02712-1)
Supplement: Supplementary file 2 — Additional file 2: Supplementary Appendix. Sensitivity Analysis. To ensure the addition of the extra 35 low educated families at waves 2 and 3 did not affect the results, we undertook a sensitivity analysis which only included families who had participated since wave 1. [file 12887_2021_2712_MOESM2_ESM.docx]

**Supplementary Appendix 2**

**Sensitivity Analysis**

Due to difficulties in recruiting families to our low educated group prior to the first wave of data collection, the decision was made to extend recruitment timelines and locations so that families could join the study at later waves. To ensure our analysis was not affected by the inclusion of additional families at each wave, we undertook a sensitivity analysis whereby we only included families who were participating at wave one. Results from the random effect model which estimate the interaction of mother’s education and wave of data collection on adult word counts, child vocalisations counts and conversational turn counts at each wave, are presented in Table S2. Figure’s S1, S2 and S3 depicts the predicted mean and 95% confidence intervals of word counts by maternal education group at 6, 12 and 18months of age. While the coefficients vary slightly compared to the total sample, the patterns of growth between waves and no meaningful differences between education groups at 6 and 12 months and a word gap emerging at 18 months is still consistent for all three measures of talk.

| **Table S2. Random effects model estimates for LENA measures across maternal education groups for sample who had participated in first wave of data collection.** | | | | |
| --- | --- | --- | --- | --- |
|  | Coef. | *p* | 95% CI | |
| **Adult Word Counts** |  |  |  | |
| *Number of adult words at 6 months among low educated = 16,768.37* |  |  |  | |
| Low Educated at 12months | -2,023.67 | 0.047 | -4,020.89, | -26.45 |
| Low Educated at 18months | -4,681.05 | 0.000 | -6,628.40, | -2,733.69 |
| High Educated at 6months | 115.20 | 0.912 | -1,927.58, | 2,157.99 |
| High Educated at 12months | -1,687.92 | 0.103 | -3,718.89, | 343.05 |
| High Educated at 18months | -149.27 | 0.884 | -2,162.08, | 1,863.54 |
| **Child Vocalisations Counts** |  |  |  | |
| *Number of child vocalizations at 6 months among low educated = 1,446.81* |  |  |  | |
| Low Educated at 12months | -33.28 | 0.652 | -178.12, | 111.55 |
| Low Educated at 18months | 313.26 | 0.008 | 82.10, | 544.43 |
| High Educated at 6months | -179.03 | 0.026 | -336.13, | -21.94 |
| High Educated at 12months | -43.06 | 0.594 | -201.37, | 115.26 |
| High Educated at 18months | 730.83 | 0.000 | 546.34, | 915.32 |
| **Conversational Turn Counts** |  |  |  | |
| *Number of conversational turns at 6 months among low educated = 348.09* |  |  |  | |
| Low Educated at 12months | 2.50 | 0.914 | -43.05, | 48.05 |
| Low Educated at 18months | 41.54 | 0.262 | -31.09, | 114.16 |
| High Educated at 6months | -23.90 | 0.234 | -63.25, | 15.44 |
| High Educated at 12months | 26.93 | 0.217 | -15.79, | 69.65 |
| High Educated at 18months | 268.21 | 0.000 | 212.68, | 323.75 |


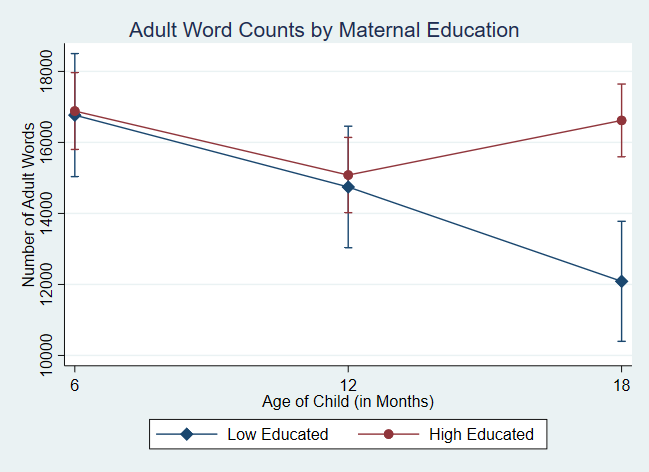


**Figure S1.** Predicted mean adult word count and 95% CI by maternal education across 6 month, 12 month and 18 month wave of data collection.


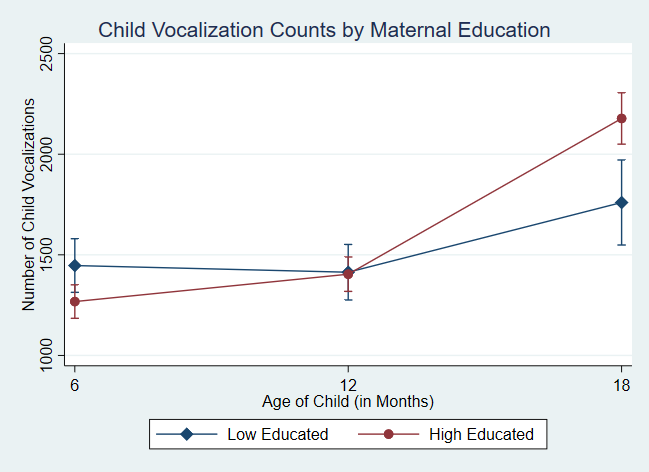


**Figure S2.** Predicted mean child vocalisation count and 95% CI by maternal education across 6 month, 12 month and 18 month wave of data collection.

**
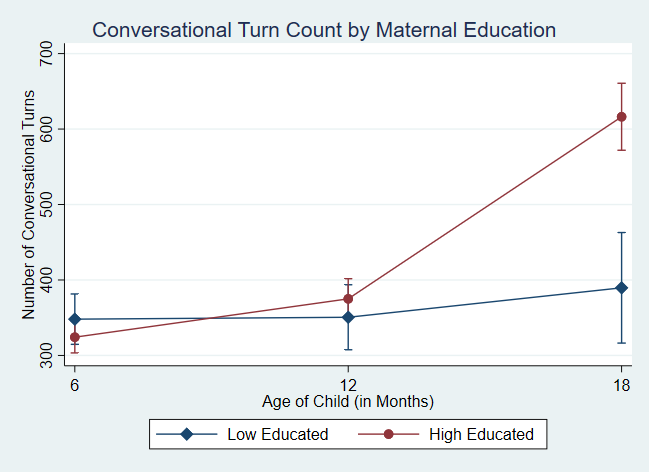
**

**Figure S3.** Predicted mean conversational turn count and 95% CI by maternal education across 6 month, 12 month and 18 month wave of data collection.
